# Estimating recent HIV incidence among young men who have sex with men: Reinvigorating, validating and implementing Osmond's algorithm for behavioral imputation

**DOI:** 10.1371/journal.pone.0204793

**Published:** 2018-10-09

**Authors:** Frits van Griensven, Philip A. Mock, Patchara Benjarattanaporn, Nakorn Premsri, Warunee Thienkrua, Keith Sabin, Anchalee Varangrat, Jinkao Zhao, Anupong Chitwarakorn, Wolfgang Hladik

**Affiliations:** 1 Thai Red Cross AIDS Research Center, Bangkok, Thailand; 2 UCSF Department of Epidemiology and Biostatistics, San Francisco, CA, United States of America; 3 Thailand Ministry of Public Health–US Centers for Disease Control and Prevention Collaboration, Nonthaburi, Thailand; 4 UNAIDS, Bangkok, Thailand; 5 Global Fund Principal Recipient Office, Department of Disease Control, Ministry of Public Health, Nonthaburi, Thailand; 6 UNAIDS, Geneva, Switzerland; 7 Global Fund to Fight AIDS, Tuberculosis and Malaria, Geneva, Switzerland; 8 US Centers for Disease Control and Prevention, Atlanta, GA, United States of America; The University of Warwick, UNITED KINGDOM

## Abstract

HIV incidence information is essential for epidemic monitoring and evaluating preventive interventions. However, reliable HIV incidence data is difficult to obtain, especially among marginalized populations, such as young men who have sex with men (YMSM). Here we evaluate the reliability of an alternative HIV incidence assessment method, behavioral imputation, as compared to serologically estimated HIV incidence. Recent HIV incidence among YMSM (aged 18 to 21 and 18 to 24 years) enrolled in a cohort study in Bangkok from 2006 to 2014 was estimated using two mid-point methods for seroconversion: 1) between age of first anal intercourse and first HIV-positive test (without previous HIV-negative test) (behavioral imputation) and 2) between the date of last negative and first positive HIV test (serological estimation). Serologically estimated HIV incidence was taken as the “gold standard” to evaluate between-method agreement. At baseline, 314 YMSM age 18 to 21 years accumulated 674 person-years (PY) of follow-up since first anal intercourse. Considering that 50 men had prevalent HIV infection, the behaviorally imputed HIV incidence was 7.4 per 100 PY. Of the remaining 264 HIV-negative men, 54 seroconverted for HIV infection during the study, accumulating 724 PY of follow-up and a serologically estimated HIV incidence of 7.5 per 100 PY. At baseline, 712 YMSM age 18 to 24 years (including 18 to 21-year-old men analyzed above) accumulated 2143 PY of follow-up since first anal intercourse. Considering that 151 men had prevalent HIV infection, the behaviorally imputed HIV incidence was 7.0 per 100 PY. Of the remaining 561 HIV-negative men, 125 seroconverted for HIV infection during the study, accumulating 1700 PY of follow-up and a serologically estimated HIV incidence of 7.4 per 100 PY. Behavioral imputation and serological estimation are in good agreement when estimating recent HIV incidence in YMSM.

## Introduction

More than thirty years into the HIV epidemic, simple, affordable and reliable methods to estimate recent HIV incidence in populations at risk are not available. Recently, this inadequacy has become particularly salient against the background of increasing HIV prevalence observed in young people, especially in young men who have sex with men (YMSM) [1]. For socio-cultural and legal reasons, collection of repeated cross-sectional and prospective serological specimens at younger ages for HIV incidence monitoring is difficult. As a result, such specimens are rarely available, especially of those younger than 21 years of age. Cohort studies provide the best measures of risk and HIV incidence, while taking in account accrual and loss-to-follow-up. In addition, such studies allow causal inference and risk factor assessment for incident HIV infection. However, cohort studies are costly, labor and participant intensive, logistically difficult to implement and sustain, and require clinical infrastructure and an enabling environment. Since incident HIV infection is a relatively rare event, large sample-sizes are needed, while infection is known to occur more often in those not-enrolled or lost-to-follow-up. Nevertheless, cohort studies are essential in guiding and powering HIV intervention studies and in monitoring the overall course of the HIV epidemic. Because of these difficulties, HIV incidence estimates are usually derived from mathematical models [2, 3], or from laboratory methods using increasing and decreasing HIV immune responses to decide on recency of infection [4]. Similarly, epidemic monitoring usually relies on cross-sectional data, such as HIV and AIDS case reports and behavioral and biological surveillance in key populations. While this information is important, uncertainty remains about recency of infection and time-order of events and often data are not age-disaggregated or those of younger ages are under-represented or ineligible. Methods for estimating HIV incidence from cross-sectional data, HIV prevalence surveys or service data have been proposed, including mathematical model-based approaches utilizing a single prevalence survey in near stable conditions [5–7]. Or more generally, incorporating historical data on changes in HIV prevalence and AIDS mortality [8], or from two prevalence surveys in the same population at two different time-points [9, 10]. A related approach is to create an artificial cohort from repeated HIV prevalence surveys along with mortality data to estimate HIV incidence while accounting for population dynamics [11]. If mortality data are not available, the use of the incubation time distribution to AIDS and death may be used as a proxy for this information [11, 12]. Another method is to utilize information from linkable repeat HIV testing and counseling attendees to create an open cohort of persons at risk for HIV infection [13–15]. Recent enhancements to laboratory testing include antibody avidity evaluation and prior HIV testing history, CD4+ cell counts and HIV RNA viral load levels to account for false-recent HIV- positives [4, 16–18]. Some of these alternative methods have been compared for agreement [10, 15]. In daily practice and outside of controlled situations, the predictive value and external validity of available HIV incidence methods have been limited and their use unsuccessful.

During the early 1990s, Osmond and colleagues [19], developed and applied an algorithm for behavioral imputation of person years (PY) of follow-up since first anal intercourse exposure to derive HIV incidence among YMSM prior to enrollment in the San Francisco Young Men’s Health Study (SFYMHS). This method, hereafter called Osmond’s Algorithm (OA), calculated the total number of PY since age at start of anal sexual intercourse and study baseline across all subjects. HIV incidence density was then estimated by dividing the number of HIV-infected YMSM at study entry by this number, times 100. While the behaviorally estimated HIV incidence prior to study entry was equal to its serologically derived equivalent during follow-up (2.6 versus 2.7 per 100 PY), follow-up time was limited (one year) and the number of accumulated PY (n = 192) and new HIV infections small (n = 5) [19]. The non-availability of prospective serological specimens from YMSM is probably one of the main reasons why OA’s reliability has not been further evaluated. Nevertheless, OA was successfully (but of unknown reliability) applied to YMSM in Amsterdam, The Netherlands in 1997 [20], in Bangkok, Thailand from 2003 to 2007 [21], in six cities in China in 2010 [22] and in adjusted fashion to YMSM across the continental United States and Puerto Rico in 2013 [23]. To assess the reliability of OA to estimate recent HIV incidence in YMSM compared to conventional prospective cohort study serology, we applied both methods in parallel against YMSM 18 to 21 and 18 to 24 years old enrolled in the Bangkok men who have sex with men cohort study (BMCS) [24–26]. As part of the BMCS, behavioral and serological data were collected simultaneously in the context of high HIV incidence in YMSM between 2006 and 2014, providing a robust number of existing and new HIV infections to evaluate agreement between the two methods.

## Materials and methods

### Study population and sampling

From April 6, 2006, to Dec 31, 2010, 1977 MSM were screened, 1764 (89.2%) were found eligible, and 1744 (88.2%) were enrolled. Of these latter men, 314 (18.0%) were 18 to 21 and 712 (40.8%) were 18 to 24 years. The goal of the BCMS was to determine prevalence and incidence of HIV infection and associated factors in MSM in preparation for HIV prevention trials. The study was conducted at the Silom Community Clinic, an integrated research and services facility for MSM, located in a Bangkok inner city hospital. Eligible men were 18 years or older, Thai nationals, residents of the greater Bangkok metropolitan area, had male-to-male penetrative anal or oral sex in the past six months, and were available for 4-monthly follow-up visits for a maximum of five years [24–26]. Men were recruited from HIV testing and counselling services provided at the Silom Community Clinic, at entertainment venues (e.g., bars, discos, saunas), through the internet, and by word of mouth. The study design has been described previously [24–26] and only methods relevant to the current analyses are presented here. The data included in this report for behavioral imputation of HIV incidence were from enrolment among YMSM 18 to 21 and 18 to 24 years. The study protocol of the BMCS was reviewed and approved by an institutional review board of the United States Centers for Disease Control and Prevention and by the Thailand Ministry of Public Health Ethical Review Committee for Research in Human Subjects. Written informed consent was obtained from all study participants.

### Estimation of serological and behaviorally imputed HIV incidence

The mid-point between the date of the last negative and first positive HIV test was taken as the estimated date of HIV seroconversion. Serologic HIV incidence was calculated by conventional prospective cohort study methodology using PY of follow-up: (the number of new HIV infections / ((the sum of the number of days elapsed since date of enrollment to the midpoint of the seroconversion interval or to the cut-off date, whichever came first / 365) x 100)). Behaviorally imputed HIV incidence was calculated according to OA by ((the number of prevalent HIV infections at baseline / the cumulative number of PY of follow-up since first anal sexual intercourse exposure prior to study entry) x 100) [19]. The earliest age at start of anal intercourse was set at the sample median of 17 years. Anal intercourse prior to that age unlikely contributed to HIV exposure, i.e., because HIV infection did not have substantial prevalence among MSM in Thailand during the corresponding calendar years [27]. In HIV-infected participants at baseline who reported a positive test date prior to study entry, the number of PY at risk was adjusted accordingly.

### Statistical analysis

Since the present study was nested in an observational cohort, it was not a-priori powered to allow comparison between serologically and behaviorally imputed HIV incidence rates in YMSM. Ninety-five percent confidence intervals (CI) for estimated serological incidence were calculated by the exact Poisson method [28]. For estimated behaviorally imputed incidence, the bootstrap method for CI construction was applied, using 10000 replications [29]. Differences between estimated serological and behaviorally imputed HIV incidence was assessed by a rate difference and significance by the 95% CI not including zero. All statistical analyses were performed using Stata v12.1 (Stata, College Station, Texas, USA).

## Results

Prior to enrollment, 314 YMSM age 18 to 21 years accumulated 674 PY of follow-up since first anal intercourse exposure. Considering that 50 men had prevalent HIV infection, the behaviorally imputed HIV incidence was 7.4 per 100 PY (95% CI: 5.4, 9.5) (Table 1). Of the remaining 264 HIV-negative YMSM of this age, 54 seroconverted for HIV infection during the study, accumulating 724 PY of follow-up and a serologically estimated HIV incidence of 7.5 per 100 PY (95% CI: 5.6, 9.7) (Table 1). Serologically and behaviorally imputed HIV incidence estimates were similar with an observed rate difference of 0.04 and not statistically significant (95% CI: -2.8, 2.9). Behaviorally imputed HIV incidence did not change after adjustment for self-reported date of HIV-positive testing (n = 3) prior to study entry. Prior to enrollment, 712 YMSM age 18 to 24 years (including men 18 to 21 years analyzed above) accumulated 2143

PY of follow-up since first anal intercourse exposure. Considering that 151 men had prevalent HIV infection, the behaviorally imputed HIV incidence was 7.0 per 100 PY (95% CI: 5.9, 8.2) (Table 1). Of the remaining 561 HIV-negative YMSM of this age, 125 seroconverted for HIV infection during the study, accumulating 1700 PY of follow-up and a serologically estimated HIV incidence of 7.4 per 100 PY (95% CI: 6.1, 8.8) (Table 1). The observed rate difference between the two age groups was 0.31, which was not statistically significant (95% CI: -1.4, 2.0).

**Table 1:** Serologically determined and behaviorally imputed HIV incidence in young men who have sex with men 18 to 21 and 18 to 24 years in the Bangkok Men who have Sex with Men Cohort Study, 2006–2014.

Method	Number of HIV infections	Number at risk	Median follow-up time (years, range)	Number of person years	HIV incidence density (95% CI^a^)
**18–21 years**					
Serological estimation	54	264	3.0 (0.0, 8.7)	724	7.5 (5.6, 9.7)
Behavioral imputation	50	314		674	7.4 (5.4, 9.5)
**18–24 years**					
Serological estimation	125	561	3.7 (0.0, 8.7)	1700	7.4 (6.1, 8.8)
Behavioral imputation	151	712		2143	7.0 (5.9, 8.2)

CI, confidence interval

^a^ Poisson exact 95% CI for serological estimates, bootstrap normal distribution derived 95% CI for behavioral estimates, based on 10,000 replications for imputation

## Discussion

In this evaluation of serological versus behaviorally imputed estimations of HIV incidence in YMSM, reliability between the two methods was established. Concordance was found among YMSM 18 to 21 years as well as among those of 18 to 24 years old. This suggests that OA may be applied to a wider age-bracket than previously done [20, 21, 23]. Our study generated robust serologically and behaviorally imputed estimates of HIV incidence. It had eight times the number of HIV sero-conversions compared to the SFYMHS, in which behavioral imputation was originally implemented [19]. Except for ascertainment of age at start of anal intercourse, commonly included as part of cross-sectional surveys among MSM, no additional data are required for behavioral imputation. This method may therefore provide a simple, cheap and reliable technique for monitoring recent HIV incidence in YMSM in settings where longitudinal data are not available.

Cross-sectional HIV prevalence to approximate HIV incidence in young populations has been applied previously to women attending antenatal care [30]. Since early mortality was low in this population, the slope of the prevalence curve could be used to estimate HIV incidence in this group [8]. However, no such institutional data are available for YMSM, who are usually marginalized with limited access to routine primary care services, including HIV testing and counseling. Other methods estimating recent HIV incidence use de-tuned laboratory assays [4, 17] to detect sequentially emerging and declining immune-responses following infection. Yet, these methods have limitations as their HIV infection window is uncertain, such as in the BED-Capture Enzyme Immune Assay (BED-CEIA) [17, 18]. Moreover, the BED-CEIA assumes stability and annualizes HIV incidence, which may not reflect reality. Additional limitations of de-tuned assays are misclassification of late-stage infections as recent, problems with “false-positives”, low-antibody titers in persons on anti-retroviral therapy (ART) and HIV-subtype dependent performance. Attempts to supplement assay-based estimates such as the limiting-antigen avidity assay with information on viral load and ART use to address the false positive problem are not perfect [31, 32]. Mathematical model approaches with one or two rounds of HIV prevalence surveys have also been proposed, but these methods assume the epidemic is in steady-state, irrespective of underlying HIV transmission dynamics [9]. There may also be unknown selection biases in sampling from one round to another, migration, or losses due to HIV-related disease and death. Methods requiring mortality or survival rates are further restricted since these are usually unknown, especially in marginalized populations. In addition, current mortality and survival data are questionable with increased access and uptake of ART over time.

The limited availability, if not absence, of HIV incidence data among MSM in lower- and middle-income countries, has become particularly poignant in the context of the ever ongoing HIV epidemic in this population [1, 33, 34]. This is particularly a problem for YMSM since these persons are at the highest risk but under parental jurisdiction, often do not have access to HIV testing and counseling and are often ineligible to consent for participation in surveillance and research. Moreover, if data are available, they are usually not disaggregated by age. As a result, HIV prevention policy and planning mostly relies on information from adults. This is particularly a problem for YMSM, as age of maturity will not protect them from HIV acquisition, especially when they are young. Our study has several limitations. It was conducted among a self-selected group of YMSM volunteers at risk for HIV infection, hence incidence estimates cannot be generalized to the YMSM population at large. Moreover, a key requirement of behavioral imputation is accurate measurement of when HIV risk begins for YMSM, which may be subject to recall bias and socially desirable answering. Here, start of exposure was measured by age instead of date of first anal sex. While start age is a solid risk-indicator, in many surveys there will be early age outliers, which are unlikely to be representative of HIV risk in YMSM. For these cases a minimum start age may be applied, such as the sample median as was done here and elsewhere [19, 21].

Thirty-five years into the HIV epidemic, simple, affordable and reliable methods to estimate recent HIV incidence in MSM are not available. Recently, this deficiency has become increasingly problematic with the expanding HIV prevalence in YMSM [1]. To stop this from happening, the HIV incidence in the youngest needs to be addressed. In this context, behavioral imputation may serve as a reliable, simple and resource friendly tool to monitor the impact of targeted biomedical and behavioral HIV preventive interventions directed at stemming the HIV epidemic among YMSM in Thailand and elsewhere.

## Supporting information

S1 Dataset (XLSX)
