# Correction: Estimating recent HIV incidence among young men who have sex with men: Reinvigorating, validating and implementing Osmond’s algorithm for behavioral imputation

**DOI:** 10.1371/journal.pone.0337128

**Published:** 2025-11-18

**Authors:** Frits van Griensven, Philip A. Mock, Patchara Benjarattanaporn, Nakorn Premsri, Warunee Thienkrua, Keith Sabin, Anchalee Varangrat, Jinkou Zhao, Anupong Chitwarakorn, Wolfgang Hladik

The eighth author’s name is spelled incorrectly. The correct name is: Jinkou Zhao.
